# Surface Chalking upon Weathering of Dark-Colored PVC Articles and Relevant Stabilizers

**DOI:** 10.3390/polym16081047

**Published:** 2024-04-10

**Authors:** Stefano Gardi, Lorenzo Giannone, Gianfranco Sarti, Gianluca Sarti

**Affiliations:** Reagens S.p.A., Via Codronchi, 4, San Giorgio di Piano, 40016 Bologna, Italy; lorenzo.giannone@reagens-group.com (L.G.); gianfranco.sarti@reagens-group.com (G.S.); gianluca.sarti@reagens-group.com (G.S.)

**Keywords:** outdoor aging, accelerated aging, chalking, dark-colored articles, acid scavengers

## Abstract

PVC items (38% carbon atoms *w*/*w*) are environmentally friendly as, unlike polyolefins (86% carbon atoms *w*/*w*), they are mainly based on chlorine, one of the most abundant elements on Earth and, so, less based on fossil resources. However, in the eco-design context, articles’ durability plays a crucial role, contributing to the enhancement of their sustainability. In this framework, the research on additives capable of increasing the weatherability of outdoor articles is essential. The theory section of the paper reviews the mechanisms of weathering that lead to PVC degradation and undermine the durability of items such as window frames or roller shutters. The weathering of PVC items is a complex phenomenon, involving photo-chemical and secondary chemical reactions, that yields the formation of conjugated polyene sequences underskin in the absence of oxygen and carbonyls in the surface. Here, the chain scission of the polymer backbone occurs, bringing about the disintegration of the surface of the item and causing the typical discoloration called chalking, especially evident in dark-colored articles. In the experimental section of the paper, the effect of different acid scavengers on item weathering has been studied using a natural outdoor and two accelerated exposures with xenon-arc and Q-UV testing devices. Results confirm that some acid scavengers are efficient in preventing chalking, but some are ineffective or even detrimental. Thus, the PVC formulations of durable articles upon weathering still depend on a complex choice of the appropriate ingredients, and several outdoor and indoor accelerated-weathering tests are needed to predict the articles’ lifetime.

## 1. Introduction

### 1.1. Scope

The durability of PVC items has gained increasing magnitude in order to save resources in the context of the eco-design of the articles [1,2]. PVC articles have a primary role in this scenario as they are made up of a sustainable material contributing significantly to energy saving [3,4]. However, despite their noteworthy durability, there is still the need to improve their stability, particularly outdoors, where degradation phenomena such as photodegradation, photooxidation, photo-catalyzed oxidation, photobleaching, side chemical reactions, and thermal degradation occur. Dark-colored articles are particularly susceptible to color change [5,6,7] and it was also considered that the colors of plastics may play a role in plastic photoaging [8]. In this context, a key role is played by the PVC stabilizers necessary to preserve the resin against degradation in processing and during items’ service life. Therefore, it is crucial to understand the chemical mechanism underlying the degradation phenomena to design more effective stabilizers. Many scientific papers have been published on the thermal degradation of PVC, and they are discussed in Refs. [9,10,11,12,13,14,15,16]. Although many studies have been published, a complete review of outdoor-weathering mechanisms leading to chalking is still missing.

This study reviews and investigates the complex mechanisms of weathering that lead to PVC outdoor degradation, particularly affecting articles like window frames and roller shutters. The study is particularly focused on the role of acid scavengers in preventing weathering drawbacks contributing to the eco-design and to enhance the sustainability of PVC items. As evident in the Section 2, the novel conclusion of this article is that, contrary to what is expected, and contrary to what normally happens in thermal stability, not all acid scavengers act in the same way for the purpose of enhancing photostability.

Thermal, photochemical, and chemical phenomena act simultaneously during the natural outdoor weathering of PVC. It is well known that PVC degrades thermally through a zip-like elimination of hydrogen chloride (HCl) with the consequent formation of conjugated-polyene sequences. Studies [11,13] have confirmed that it acts through an ion-pair mechanism where the free radicals generated by the interaction between HCl and polyene sequences play a crucial role [17].

The theory section of this paper describes the weathering mechanisms in detail and step by step. The Section 2 has the aim of studying the impact of some acid scavengers on the extension of the durability of dark-colored PVC articles upon weathering and, specifically, their impact on chalking. To this latter aim, natural- and accelerated-weathering tests are used to confirm the reliability of fast methods to screen the appropriate stabilizer recipes.

### 1.2. Theory

#### 1.2.1. Introduction

As PVC is only transparent at wavelengths of more than 250 nm, the initiation is likely due to chromophores found in the PVC resin. However, polyenes rapidly become the main absorbing species among chromophores due to their high extinction coefficient [18]. Only polyene sequences with more than four double bonds can interact with sunlight at the ground, as the atmosphere screens wavelengths shorter than about 290 nm (Table 1, [19]).

When photons hit the PVC item, two kinds of reactions take place. The first generates conjugated polyene sequences and HCl [18]. The chromophores absorb the photons, and one of the non-radiative pathways releases Cl radicals, starting the zip-elimination quickly before oxygen can become involved [18]. This yields conjugate-polyene sequences and their crosslinking. The UV screen from polyene sequences protects the matrix from further degradation.

**Figure 1:**
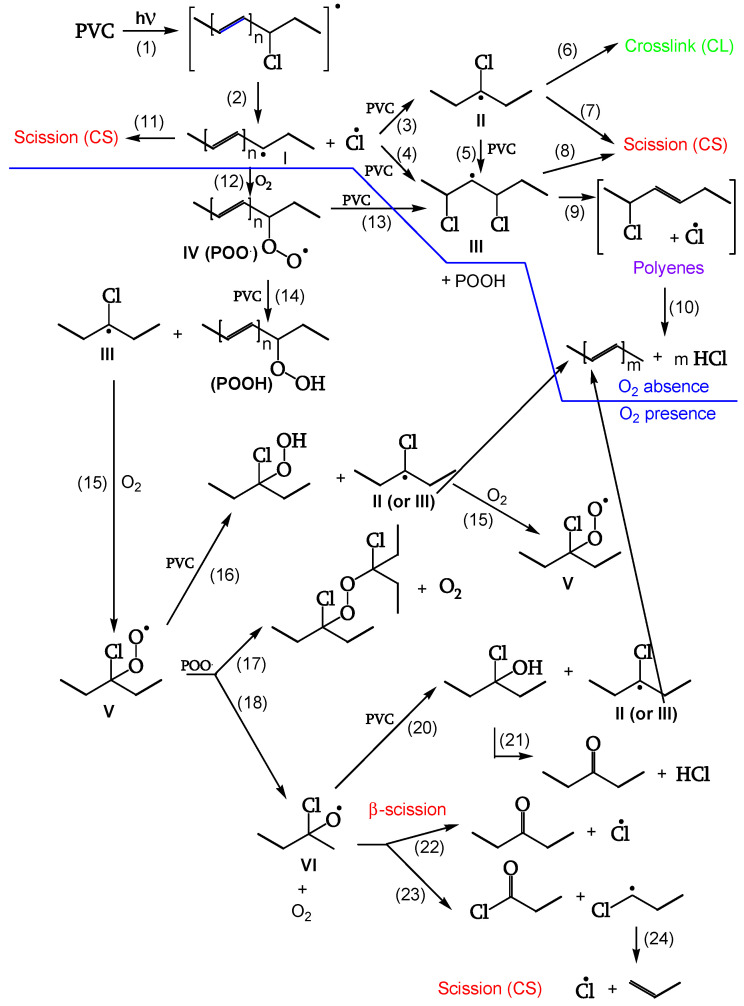
Overall photodegradation of PVC, ref. [18].

**Figure 2:**
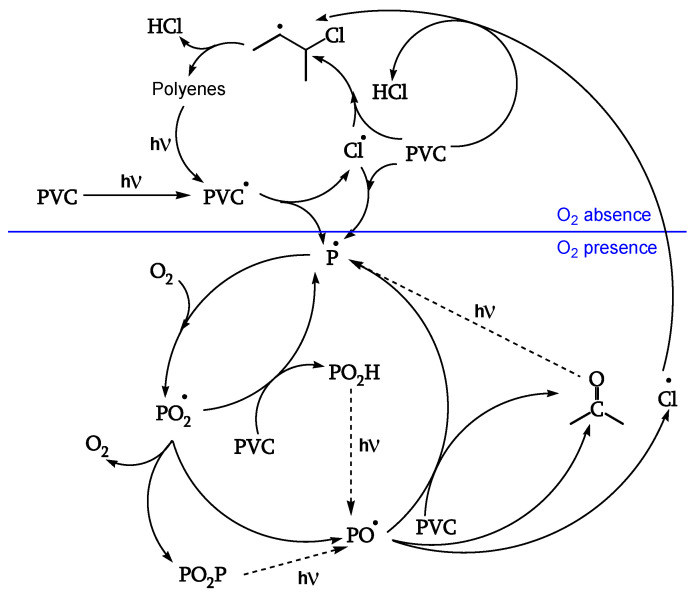
Overall photodegradation of PVC (─── first stage; - - - second stage), ref. [18].

A second series of reactions involves oxygen, and it causes a severe photo-oxidation, bringing about chain scission and the formation of carbonyls (Figure 1 and Figure 2). Zip elimination will dominate where oxygen is lacking. Therefore, it will take place underskin and is called photodegradation. The second series of reactions will take place in the proximity of the surface, where oxygen concentration is high; it is called photo-oxidation (Figure 1 and Figure 2). Titanium dioxide can promote a strong photo-oxidation, and this is called photocatalyzed oxidation. Fillers, commonly found in the PVC compound, can react with HCl, impacting the weatherability of the article. When the photo or photocatalyzed oxidation destroys the surface, it exposes the photodegraded underskin to light and oxygen, and the polyene sequences are oxidized through photobleaching [20].

Figure 1 outlines the overall first stage of the PVC photodegradation and photo-oxidation mechanism leading to chain scission, polyene sequence formation, and crosslinking, depending on the exposure conditions [18].

As is evident from the calculated quantum yields (Table 2), the presence of oxygen also enhances chain scission and triggers the generation of peroxides and ketones [18].

The reactions of Figure 1 are also described in Figure 2 in a simplified form with complete lines. This latter Figure, however, also outlines two subsequent degradation stages.

In fact, the reaction goes further in a second stage (dashed lines in Figure 2), leading to photocleavage of peroxides and ketones and generating further initiating radicals [18].

The following paragraphs will detail the chemistry of photodegradation and photo-oxidation.

#### 1.2.2. Photodegradation

##### Initiation

**Figure 3:**
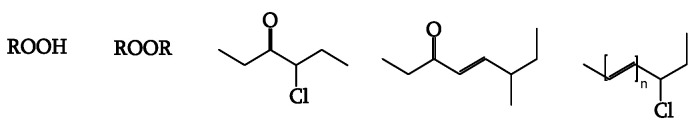
Possible chromophores.

Impurities and chromophores trigger photodegradation. Polyenes, hydroperoxides, and ketones are possible chromophores (Figure 3).

**Figure 4:**
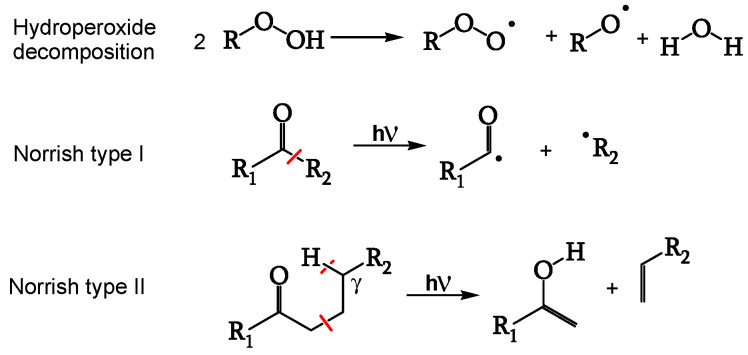
Chromophores’ reactions, ref. [21] (red lines are bond scissions).

Hydroperoxides generate radicals upon light irradiation (Figure 4, [21]) as efficiently as ketones do through the Norrish type I reaction (Figure 4, [21]). Polyene sequences, having a high extinction coefficient, are the most absorbent chromophores [18]. Table 1 [19] gives the UV-visible absorptions of polyenes of different lengths.

Moreover, photodegradation studies in the presence and absence of oxygen showed the independence of the quantum yield of HCl on the irradiation time and the initial amount of unsaturation. That leads to the hypothesis of alkene-photo-sensitized degradation processes [18,22].

**Figure 5:**
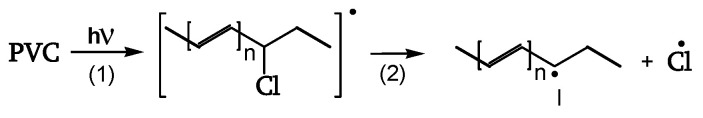
Initiation.

In these processes, excited singlet polyenes deactivate by radiative and non-radiative processes. Only 1% of absorbed photons generate HCl due to the restricted mobility that favors radical recombination (reaction 2 of Figure 5) [18].

##### Polyene Formation

**Figure 6:**
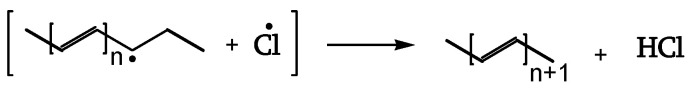
Cl• Cage reaction—polyene formation.

Cage reaction of Cl• with the adjacent CH_2_ leads to termination and HCl evolution (Figure 6).

**Figure 7:**
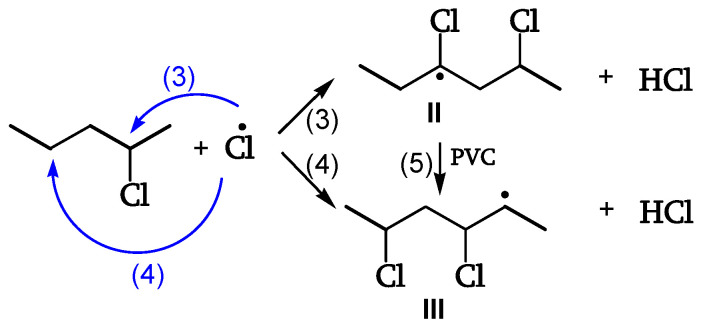
Cl• Out-of-cage reaction—hydrogen abstraction.

Cl• escaping the cage abstracts H from CHCl (reaction 3) or, preferably (five times faster), from CH_2_ (reaction 4). The unstable radical (II) yields the α-radical (III) (Figure 7, [18]).

**Figure 8:**
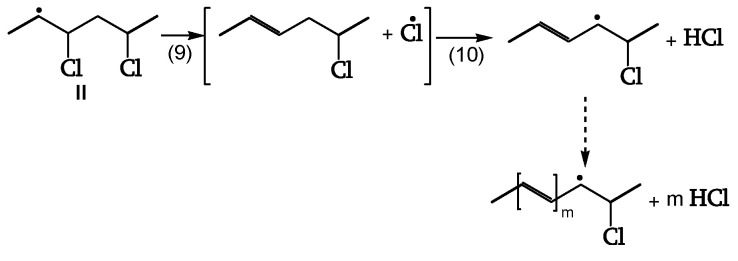
Polyene chain increase.

The β-chloroalky radical (II) splits off a Cl• (reaction 9). That Cl• (cage reaction 10) reacts with the close-by CH_2,_ initiating the dehydrochlorination, generating polyenes of increasing length (dashed line) (Figure 8, [18]).

##### Chain Scission and Crosslinking

**Figure 9:**
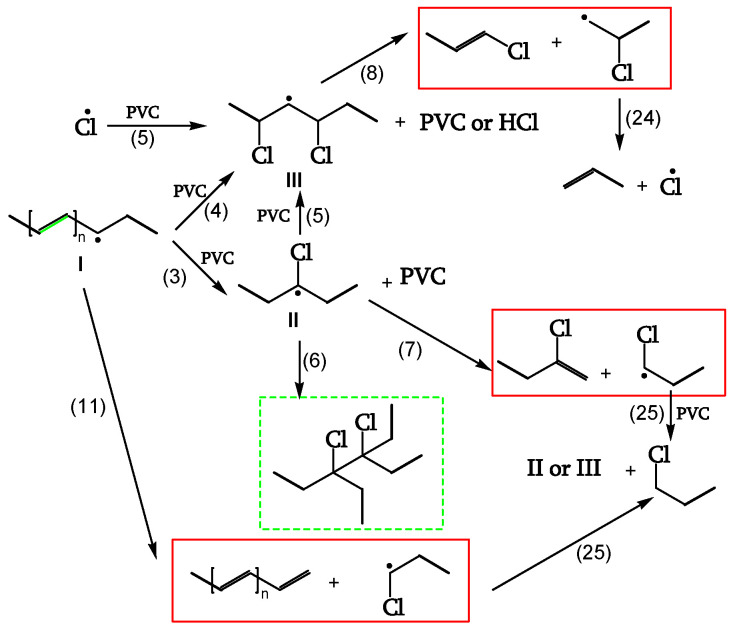
▬ Chain-scission reactions, --- Crosslinking reaction.

Radicals can be stabilized by β-scission (chain scission) of C-C bonds (reactions 7, 8, 11). The released radical fragments (•CHCl-CH_2_- or •CH_2_-CHCl-) propagate either by reacting with PVC to regenerate II and III (reaction 25) or by splitting off a Cl• (reaction 24). Radical III is sufficiently stable and provides crosslinking (reaction 6) (Figure 9, [18]).

#### 1.2.3. Photooxidation

##### Initiation

The same initiation occurs in the presence or absence of oxygen, as the excited singlet polyenes (with very short lifetimes, about 10^−9^ s) cannot be quenched by it (Figure 5, [18]).

##### Peroxidation

**Figure 10:**
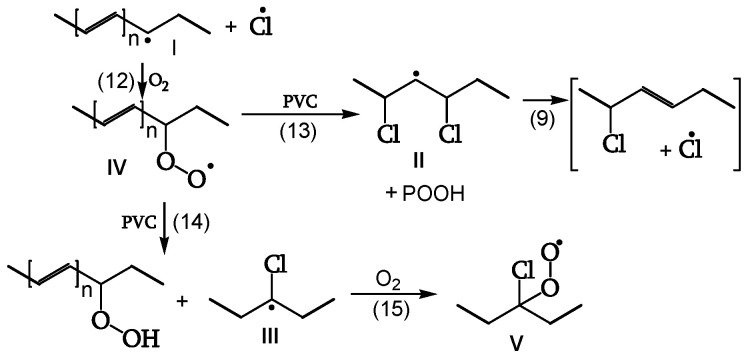
Peroxidation.

The polyenyl radical I (contrary to the less stable radical II) reacts with oxygen (reaction 12) to give peroxy radical IV. The attack on CH_2_ of the PVC chain yields radical II (reaction 13). The attack on the CHCl of the PVC chain yields radical III (reaction 14). Radical III does not have labile β-Cl and reacts with oxygen to give γ-chloroalkylperoxy radical V (reaction 15) (Figure 10, [18]).

##### Reaction of the Peroxy Radical

**Figure 11:**
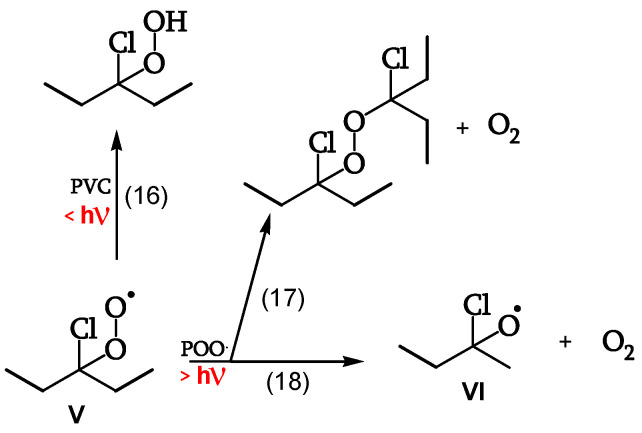
Peroxy-radical reactions.

At low irradiations, radical V abstracts H from PVC (reaction 16). The same radical V, at higher irradiations, either reacts with another peroxy radical to give “termination” (reaction 17) or reacts with another peroxy radical to give alkoxy radicals VI (reaction 18) (Figure 11, [18]). Peroxide obtained by reaction 17 is a photoreactive re-initiating site.

##### Reaction of the Alkoxy Radicals

**Figure 12:**
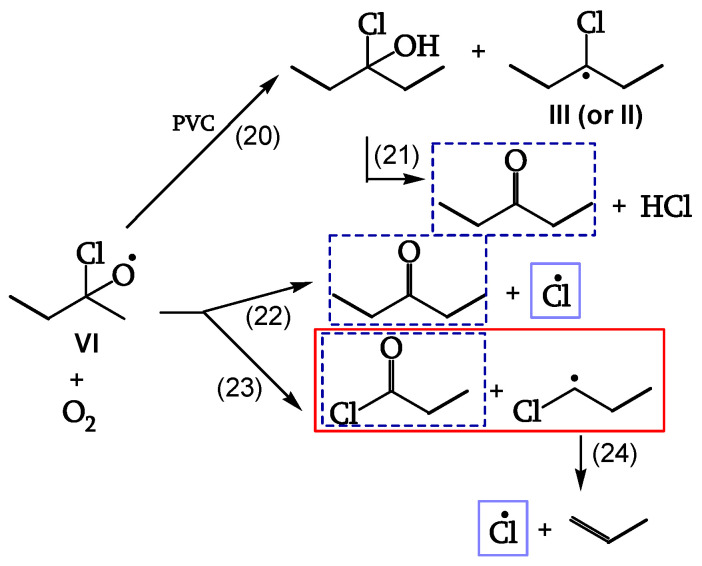
Alkoxy-radical reactions: ▬▬ Chain-scission reactions, ▬ ▬ ketone generation, ▬▬ chloro-radical generation.

Alkoxy radical VI either abstracts H from PVC (reaction 20), eventually decomposing to the corresponding ketone (reaction 21), or is stabilized by β-scission, giving ketones and either Cl• (reaction 22) or chain scission (reaction 23). These additional Cl• account for a 40% increase of θ _HCl_ in the presence of oxygen (Figure 12, [18]).

##### Termination

**Figure 13:**
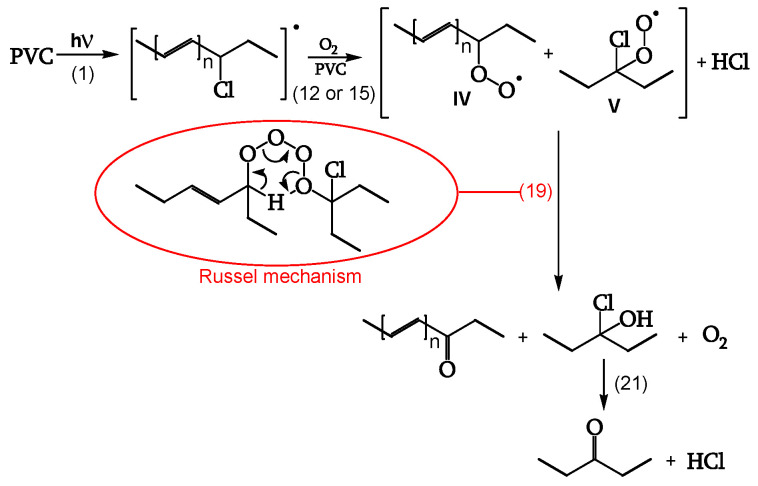
Termination.

Cage reaction between peroxy radicals IV and V, via a Russel mechanism (reaction 19), leads to a ketone and an α-chloroalcohol. The α-chloroalcohol decomposes to give a ketone (reaction 21). That is why θ _C=O_ > θ _POOH,_ as evidenced in Table 2 (Figure 13, [18]).

#### 1.2.4. Photobleaching

**Figure 14:**
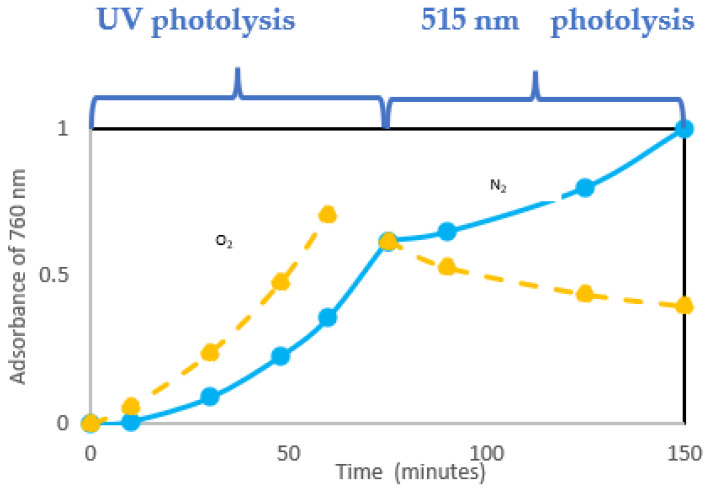
Variation of absorbance with UV irradiation (**left**) and 514.5 nm photolysis (**right**) with and without oxygen, ref. [20].

Experiments in Ref. [20] were carried out, irradiating samples of PVC (with and without oxygen) with a UV lamp and then irradiating them with a lamp emitting in the solar spectrum region (i.e., 515 nm). In this second stage, a lowering of the absorbance was attributed to the photobleaching of conjugated double bonds by oxygen: in fact by far counterbalances double bond formation (Figure 14).

**Figure 15:**
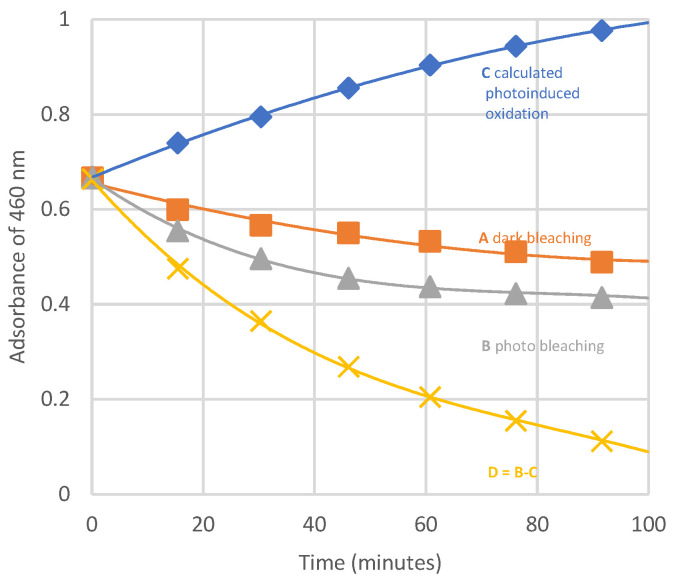
Kinetics of oxygen bleaching. A: dark bleaching, B: photobleaching, C: calculated increase of absorbance due to polyene formation, D: calculated neat photobleaching (=curve B–curve C), ref. [20].

This kind of bleaching also occurs in the dark, and Figure 15 shows that the neat contribution of photobleaching is reduced by the absorbance increase due to polyenes formation.

**Figure 16:**
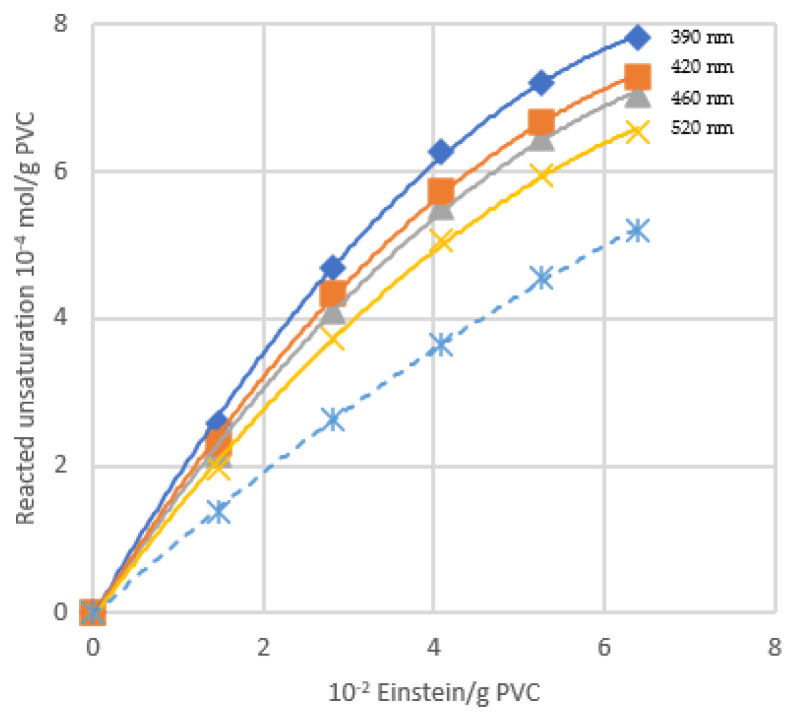
Amount of double bond reacting with oxygen vs. number of photons absorbed at various wavelengths (; x HCl evolved, ref. [20]).

Figure 14 shows the extent of photobleaching (full lines) vs. dehydrochlorination (dashed line) under photolysis. The quantum yield of photobleaching is higher than dehydrochlorination one (θ _photobleaching_ = 1.5 × 10^−2^ vs. θ _HCl_ = 0.9 × 10^−2^). Θ _photobleaching_ is not very dependent on the wavelength. Thus, it is similar regardless of the length of the conjugated-polyene sequence (as will be schematically described in Figure 16).

**Figure 17:**
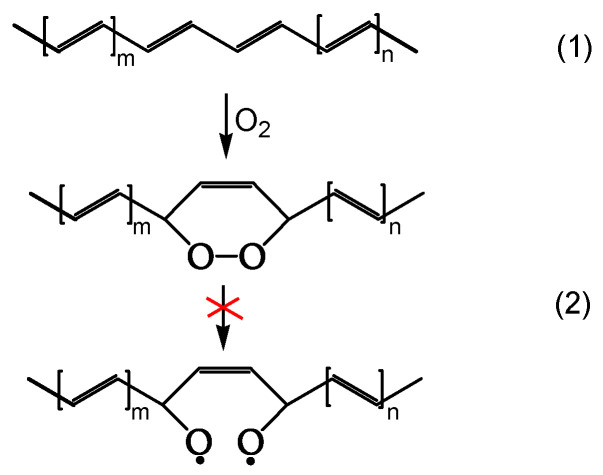
Cyclic peroxide formation, ref. [20] (red mark means that the reaction is not happening).

The mechanism leading to dark bleaching involves a step where ground-state oxygen reacts with polyenes (reaction 1 of Figure 17), generating stable cyclic peroxides at room temperature. Their stability stems from very little HCl having evolved during oxygen bleaching in the dark.

**Figure 18:**
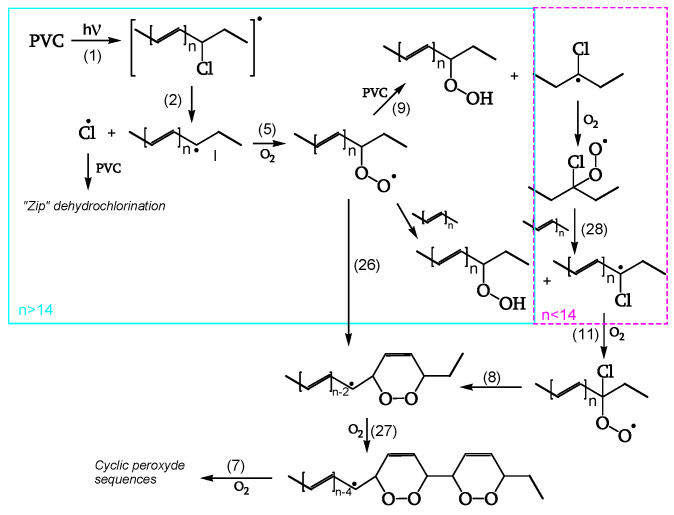
Photobleaching by oxygen of short (**- - -**) and long (▬▬) polyene sequences, ref. [20].

Unlike dark-bleaching, in photobleaching (Figure 18), polyenyl radical I is stable enough to react with oxygen (reaction 5). The resulting polyenyl peroxy radical may form cyclic peroxides (reaction 26). Then, a step-by-step polyene sequence-shortening follows, forming adjacent cyclic peroxides (reactions 7, 27). Reactions 26 and 27 also apply to shorter polyene peroxy radicals resulting from reactions 5, 11, and 28, thus accounting for the bleaching of polyenes with *n* < 14 (not absorbing the 515 nm radiation).

#### 1.2.5. Segmentation of Degradation Layers

**Figure 19:**
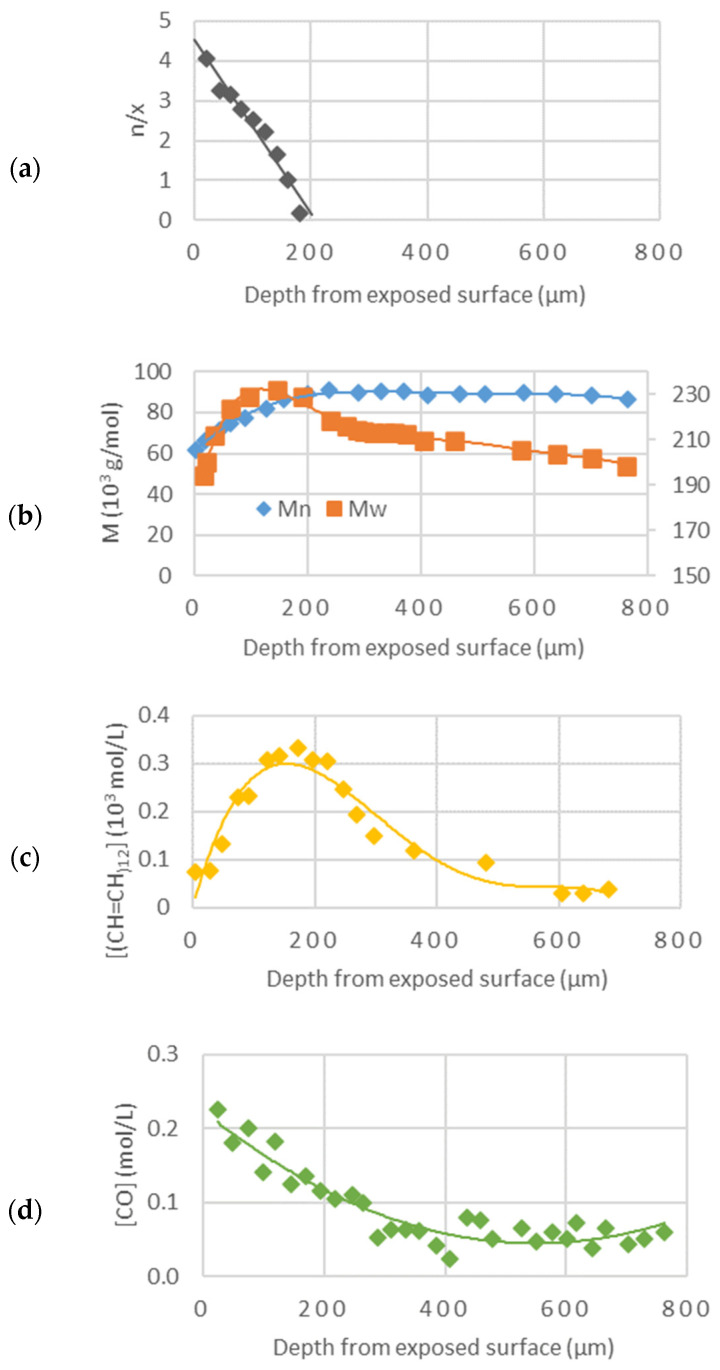
Synoptic comparison along the thickness of the sample during photodegradation: depth distribution of chain scission *n* over crosslink x ratio (**a**); depth distribution of average molecular weight Mw and Mn (**b**); depth distribution of polyene concentration (**c**); depth distribution of carbonyl concentration (**d**), ref. [24].

Studies on the generation of polyenes and carbonyl species in the presence of stabilizers have been performed and led to the conclusion that the (photo)oxidation is diffusion-controlled [23]. This concept was completed by further studies on crosslink/chain-scission ratio, molecular weight, presence of polyenes, and carbonyl species along the thickness of an article exposed to sunlight (Figure 19, [24]).

**Figure 20:**
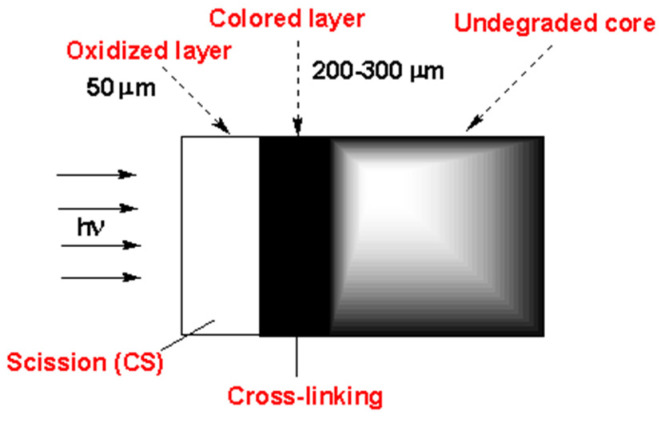
Segmentation of degradation layer.

That experiment showed that there are three zones (roughly indicated by the different colors in Figure 20) along the thickness of a PVC article exposed to sunlight:

1. A superficial zone (about 50 microns thick) with the predominance of oxidation products and chain scission: oxidation is oxygen diffusion-controlled in this region; chain scission results from oxidation reactions and is followed by chalking.
2. A lower underskin zone (between 50 and 300 microns) with a predominance of conjugated polyenes. This region is dominated by crosslinking (resulting from C• radicals) and polyene growth.
3. An undegraded core zone beyond 300 microns. In this region, photochemical reactions do not occur as the photons are screened by polyenes when they are increasingly generated after a specific time.

**Figure 21:**
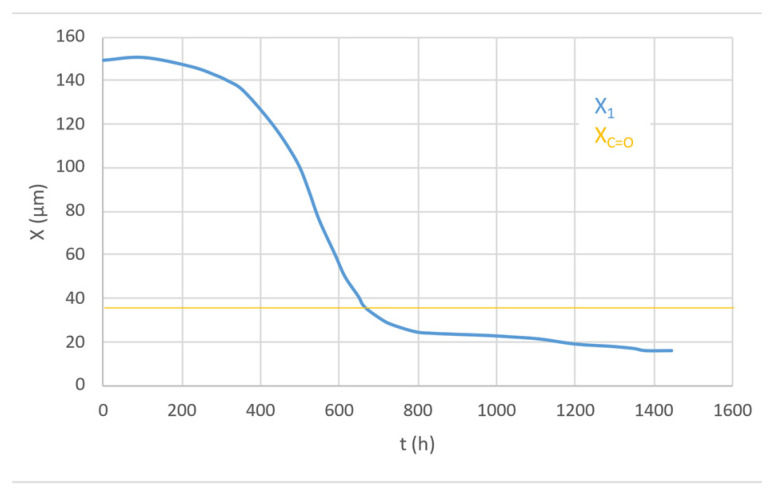
Change of the thickness of irradiated layer X_1_ and oxygenated layer X_co_, ref. [25]. X_1_ = thickness where 90% of the irradiation is absorbed.

The formation of the two superficial degraded layers occurs because:

1. At the beginning of the exposure, the irradiation layer (X_1_) is higher than the diffusion-controlled Oxidation layer (Xco)
2. Later on, polyene build-up provides a screen effect, and the irradiation layer (X_1_) becomes lower than the oxidation layer (Xco); this prevents any progression of the oxidation-front towards the core (Figure 21, [25]).

1. At a certain point in time, the superficial layer is embrittled by oxidative chain scission and cracks producing chalking that is, naturally, mechanically removed from the surface.
2. The polyenes, lacking their «protective layer», undergo photobleaching, shifting the boundary between degraded zones towards the core.

The sequential generation of dark polyene sequences, their bleaching, and eventually the erosion of the cracked surface triggers the cyclic change of color typical in outdoor exposure [26,27].

The thickness of the oxidation profile (X_co_) increases with higher temperatures due to the higher diffusivity of oxygen, while it is independent of irradiation intensity. The depth of the max polyene concentration decreases with higher irradiation intensity and increases with higher temperature.

As the outer layer of PVC degrades, particles of pigment like titanium dioxide and fillers like CaCO_3_ are released, producing a white powdery deposit; this phenomenon is known as “chalking.” It is known that different colors may affect plastic photoaging by influencing its solar absorbance [8]. In addition, as chalking makes the article look whitish, this phenomenon is more apparent in dark-colored samples than in the white-pigmented ones. This particular weakness of dark-colored articles is well known and is a genuine challenge for manufacturers [5,6,7].

#### 1.2.6. Effects of Pigments and Fillers

##### Effects of Titanium Dioxide Pigments

**Figure 22:**
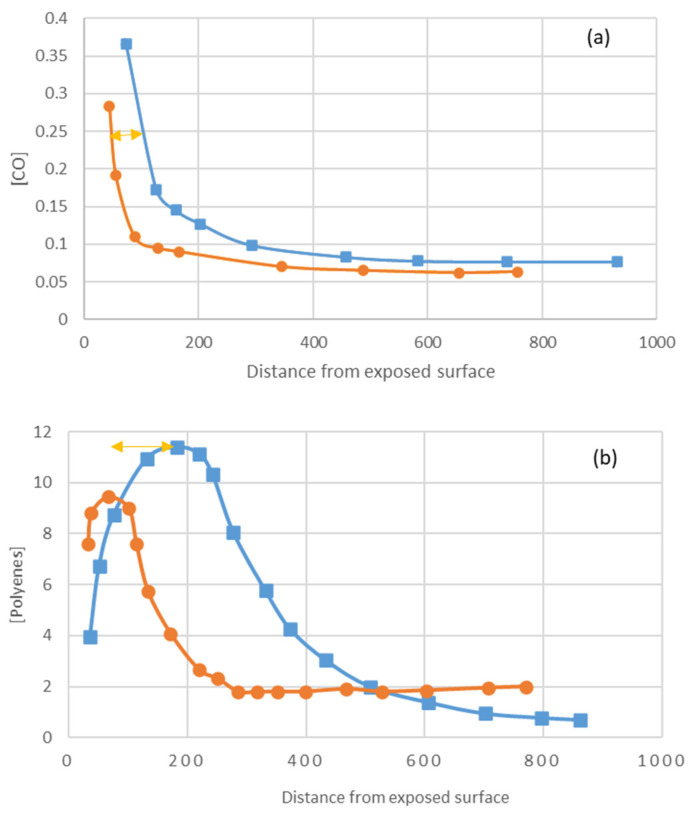

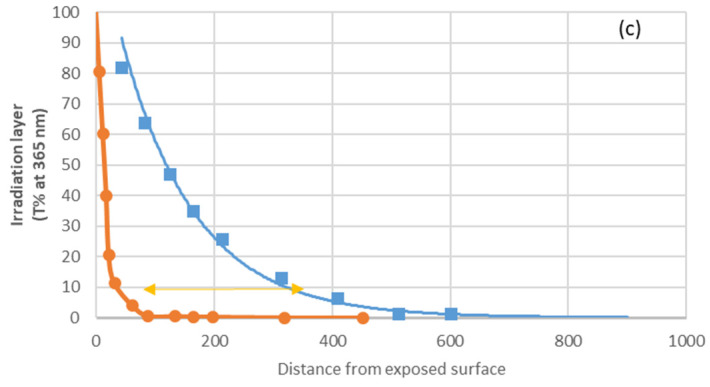
Degradation product profile for carbonyl and polyenes ((**a**,**b**), respectively) and irradiation profile (**c**). Blue squares: with titanium dioxide, Orange dots: without titanium dioxide, ref. [28] (yellow arrows indicate the difference in distance from the surface of the variable in function of the presence or absence of titanium dioxide).

Figure 22 shows that titanium dioxide limits light penetration to 1-fifth. (Figure 22c). The fact that the thickness of the degraded layer is 1-third (Figure 22a for carbonyls, b for polyenes) [28] is due to the following reasons:

1. UV light penetration decreases with time as polyenes build up.
2. Small radicals such as OH● and Cl● diffuse beyond the irradiation layer.
3. Photoreactions can be initiated by wavelength radiation close to the titanium dioxide cut-off (i.e., the limit between transmittance absorption and scattering, 365 nm).

In addition, it is also well known that, despite its screening effect, titanium dioxide (if not adequately coated or in the anatase form) promotes polymer degradation [29]. Furthermore, due to the PVC surface layer’s degradation, titanium dioxide contributes to the chalking phenomenon described above, in the presence of water, as usually it happens in hot, humid climates such as Florida. On the other hand, chalking is not a common issue in hot, dry climates. This behavior is due to the typical mechanism through which TiO_2_ brings about the oxidation of the organic substances in the presence of water [18,30].

##### Effects of Fillers

Fillers also influence the degradation and chalking of weathered PVC articles. CaCO_3_ is an example of a filler capable of reacting with the HCl from PVC degradation to give the water-soluble calcium chloride. Its washout leaves holes, increasing the surface area subject to degradation and worsening the weathering. This phenomenon is increasingly evident if the filler amount rises [31].

#### 1.2.7. HCl Effect on Carbonyl Formation and Inhibition

**Figure 23:**
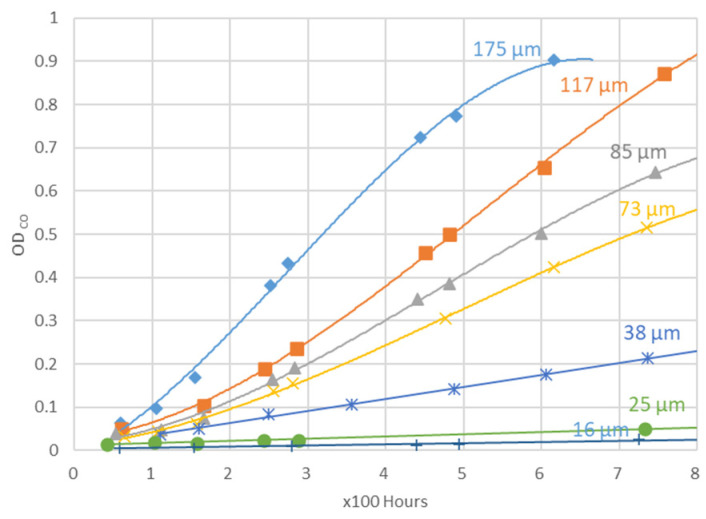
Optical density (OD) of carbonyl peak vs. exposure times for films at a different thickness (16 microns—dark blue crosses-, 25 microns— green dots, 38 microns— blue stars, 73 microns— yellow crosses, 85 microns— grey triangles, 117 microns— orange squares, 175 microns—blue diamonds -), ref. [32].

Schemes of Figure 1 and Figure 2 do not involve HCl in the reactions in the presence of oxygen. However, it was observed that, in PVC photooxidation, there is a first autocatalytic phase in the formation and a subsequent inhibition phase for carbonyl species (Figure 23, [32]). As evident at short and long irradiation times, the initial auto-acceleration and the final auto-inhibition are favored when the thickness is high: the diffusion of a reactant from the sample to the atmosphere can thus control their kinetic behavior.

**Figure 24:**
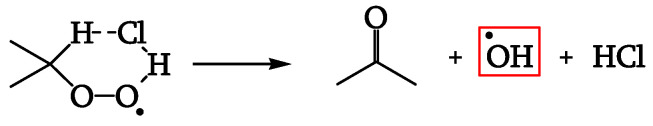
HCl-catalyzed hydroperoxide decomposition, ref. [32].

This reactant may, thus, be a gas such as HCl, which can explain the initial auto-acceleration with hydroxyl radical formation (Figure 24).

**Figure 25:**
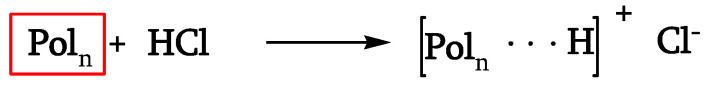
HCl inhibition of polyene reactivity, ref. [32].

HCl can also explain the final auto-inhibition. A charge transfer complex of HCl with polyenes (not photochemically active, different from polyenes themselves) is described in the literature (Figure 25).

## 2. Experimental

As degradation generates cracking and the final effect is the chalking of the surface, this mainly affects the color variation of dark-colored articles. For this reason, several dark-green samples were prepared, as this color is particularly affected by weathering.

In the presence of pigments with high light fastness, the color change is due mainly to the chalking from the degraded surface rather than the degradation of the pigment itself. As shown above, carbonyl formation is limited to the surface as well (Section 1.2.5). Therefore, as HCl seems to play a role in the carbonyl formation (Section 1.2.7), samples with an increasingly higher loading of acid scavengers were prepared to study their effect on surface degradation of the dark-green-colored samples. Several acid scavengers are available and among them are hydrotalcites (also exchanged with different metals such as zinc, lithium, tin, titanium, and zirconium), hydrocalumite, ettringites, dawsonites, garnets, calcium and magnesium hydroxides, zeolites, metal carboxylates, organic phosphites, and epoxy compounds. Their general mechanism of action is well known and reviewed in the literature [33]. 

### 2.1. Materials and Methods

Samples are manufactured using a base formulation made up of 100 parts *w*/*w* of PVC K65 from Vynova (Tessenderlo, Belgium) (about Mw of 81 × 10^3^ g/mol and Mn of 41 × 10^3^ g/mol), 15 parts *w*/*w* of calcium Carbonate Valtochim from Umbriafiller (Nocera Scalo, Perugia, Italy), 0.25 parts *w*/*w* of acrylic processing aid Reamod P220 from Reagens (San Giorgio di Piano, Bologna, Italy), lubricants REALUBE RL/105 CP from Reagens (San Giorgio di Piano, Bologna, Italy) (0.3 parts *w*/*w*), REALUBE SS 16-18 from Reagens (San Giorgio di Piano, Bologna, Italy) (0.2 parts *w*/*w*), REALUBE PO from Reagens (San Giorgio di Piano, Bologna, Italy) (0.15 parts *w*/*w*), Calcium-Zinc Corepack (1.8 parts *w*/*w*) from Reagens (San Giorgio di Piano, Bologna, Italy). V5000179 precolor PVC Dark green Cool Type from MASTER TEC GmbH (Oberhaid, Germany) is the selected pigment, has a high light fastness and contains titanium dioxide.

### 2.2. Sample Preparation

The formulations in Table 3 were prepared by adding the indicated acid scavengers to the base formulation, prepared as described above, into a dry blend obtained using a PlasMec (Lonate Pozzolo, Varese, Italy) laboratory high-speed heater and cooler mixer combination. A sample without any acid scavenger is not presented as, in its absence, PVC samples would not have been obtained, as it is a key component of thermal stabilizers too.

The following protocol was used for the blending:

- Ambient temperature: Start low-speed, add all components, and switch to high speed
- 110 °C: Discharge the heater mixer into the cooler mixer
- 40 °C: Discharge cooler mixer

All specimens (cut into pieces of 2 bv 10 cm) have been produced by a Bausano (Rivarolo Canavese, Torino, Italy) MD30 fully instrumented parallel twin-screw extruder with the following parameters:

- Temperature profile: 145, 150, 160, 170, 165, 190, 195 °C
- Screw speed: 22 rpm
- Torque: about 21–22 Nm
- Mass Temperature: about 180 °C

### 2.3. Weathering

#### 2.3.1. Natural Outdoor Exposure

Natural outdoor exposure was carried out at the Reagens plant in San Giorgio di Piano (BO, Italy), southward 45°. San Giorgio di Piano is characterized by:

- A typical annual solar irradiance of around 5.40 GJ/m^2^ (Registered by Dexter Arpae San Pietro CapodiFiume available at https://simc.arpae.it/dext3r/, access date 13 November 2022).
- The average temperature of the warmest month of the year being 25 °C (Registered by Dexter Arpae San Pietro CapodiFiume available at https://simc.arpae.it/dext3r/, access date 13 November 2022).
- Severe climate, according to DIN EN 12608-1:2020 [34].

#### 2.3.2. Accelerated Exposure

Accelerated exposure was carried out both with a xenon-arc tester Altas (Mount Prospect, Illinois, USA) Ci4000 Weather-Ometer according to DIN EN 513:2018 [35], method 2 (hot climate—Irradiation 12 GJ/m^2^, wavelength 280–800 nm, black panel temperature 65 °C, 114 min dry/6 min water spray, RH dry cycle 65%) and with a Q-Lab (Westlake, Ohio, USA) QUV Accelerated Weathering Tester according to ISO 4892-3:2016 [36] Method A/Cycle 1 (Irradiation 0,76 Wm^2^ at 340 nm, UVA 340 (Type 1 A), black panel temperature 50 °C).

### 2.4. Color Measurement

Color has been measured on three replicates with an X-Rite SP 62 colorimeter working at D65/10. Evaluations were based on Delta E, representing the overall color change. The definition of Delta E and L*, a*. and b* is in DIN EN 13245-1:2010 [37].

## 3. Results and Discussion

**Figure 26:**
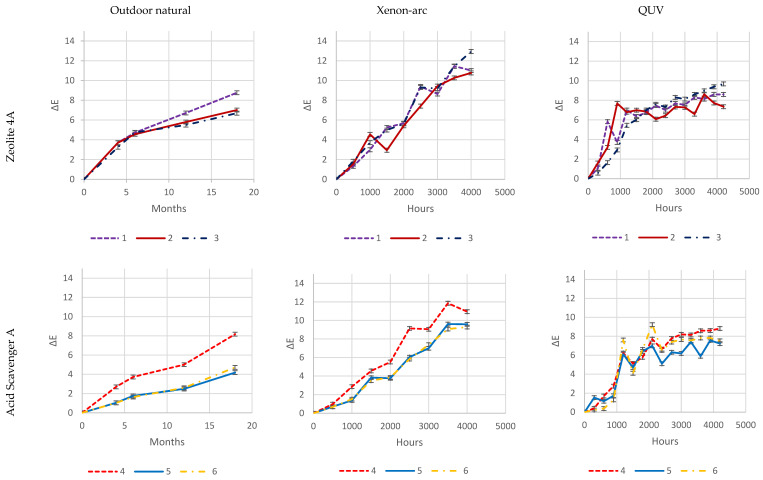

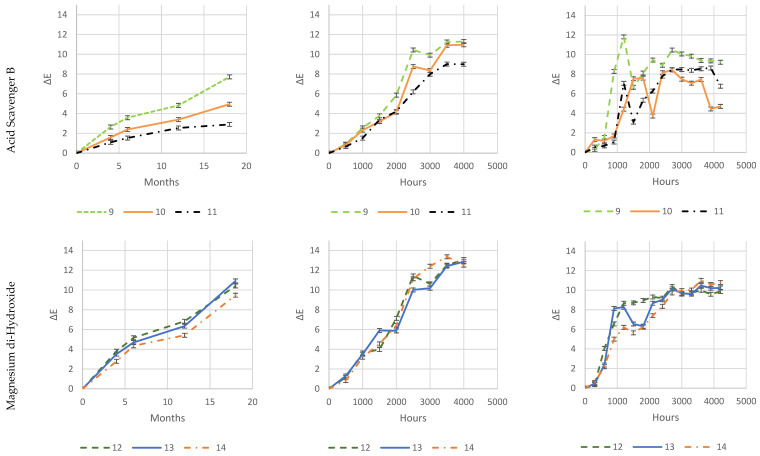

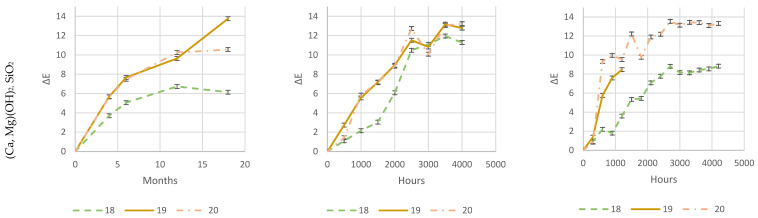
Color variation (Delta E) upon natural outdoor, accelerated Xenon-arc, and accelerated Q-UV exposure of samples in the function of different acid scavengers (listed in Table 3) at increased loadings.

As it is evident from Figure 26, both natural-outdoor and accelerated-weathering data are in accordance and show that higher loadings of some acid scavengers are more effective than others against photodegradation (e.g., Acid Scavenger One-pack A and Acid Scavenger One-pack B), while some of them are almost ineffective (e.g., simple Magnesium di-hydroxide, or simple Zeolite 4A), or even detrimental as a trivial (Ca, Mg)(OH)_2_, SiO_2_.

Increased loadings of Zeolite 4A and, in particular, Magnesium di-hydroxide, has no impact on color change during exposure, which is evident in all three exposure types.

An increased loading of (Ca, Mg)(OH)_2_, SiO_2_, is even increasingly detrimental for the color change when it raises from 1 phr to 4 or 8 phr. This is probably due to the reaction of HCl with calcium, generating water-soluble CaCl_2_. As in the case of CaCO_3_, outlined in Section 1.2.6, CaCl_2_ brings water into the matrix and makes the surface more prone to photo-oxidation. Furthermore, it is washed out during natural outdoor rain times and in accelerated-weathering devices during their wet cycles, leaving holes, cavities, and an uneven surface. Thus, the increased surface area and a stronger photo-oxidation worsen the weathering (Section 1.2.6). As in the case of CaCO_3_, this phenomenon is increasingly evident as the amount rises. This adverse effect probably offsets the beneficial HCl scavenging mechanism.

Zeolite 4A provides slightly higher protection at 4 and 8 phr, while an increase in Magnesium di-Hydroxide loading does not.

On the contrary, well-balanced acid-scavenger packages like Acid Scavenger One-Pack A and Acid Scavenger One-Pack B provide increasingly stronger protection against weathering as their loading increases. In particular, Acid Scavenger One-Pack B provides the strongest protection effect, lowering Delta E when it is increased from 4 to 8 phr. On the contrary, Acid Scavenger One-Pack A effect is leveled off at a concentration above 4 phr.

It is noteworthy that milder (and closer to reality) weathering methods allow for better discrimination between the performance of formulations than accelerated ones do. For example, this is evident for Acid Scavengers One-Pack A where the difference in performance between its 2 and 4–8 phr is well evident in natural weathering. On the contrary, it is less noticeable in the more aggressive xenon-arc tester, and almost not evident in the far more aggressive QUV tester. Regarding Acid Scavenger One-Pack B, natural weathering well discriminates among the performance of its 1, 4, and 8 phr loading. This gap is narrower in xenon-arc tester where the difference between 1 and 4 phr is negligible. In the QUV test apparatus, the Acid Scavenger One-Pack B loading effect is not evident at all. The slightly improved protection of Zeolite 4A at higher loading is not evident at all, not only in the most accelerated QUV tester, but also in the intermediate accelerated xenon-arc tester.

This emphasizes the importance of extended testing in the real field for a better understanding of the weathering performance, while accelerated-weathering tests should be used only for screening purposes. From a practical point of view, an accelerated screening QUV test would have been helpful to discriminate between evidently detrimental additives (e.g., (Ca, Mg)(OH)_2_, SiO_2_) and beneficial ones (e.g., Acid Scavenger One-Pack B), but only natural outdoor exposure has been used to investigate the loading effect of the latter.

## 4. Conclusions

Acid scavengers are remarkable substances designed to prevent thermal degradation and scavenge HCl during the thermal decomposition and combustion of low-acidity PVC compounds for cables [38,39,40]. For these reasons, they already are crucial ingredients in formulating PVC compounds.

This paper shows another, somehow new aspect of some acid scavengers in preventing the drawbacks of exposure of the items to the outdoors, where thermal degradation and photodegradation, photooxidation, photo-catalyzed oxidation, and chemical reactions bring about phenomena affecting not only the aesthetic but also the mechanical properties of the articles.

The comprehensive understanding of the photodegradation mechanism of PVC gained from the experiments carried out proves that acid scavengers not only are effective against the HCl-catalyzed zip-like elimination that proceeds in the absence of oxygen but also against carbonyl formation, chain scission, and surface chalking in the presence of oxygen.

This new experimental evidence confirms the mechanism outlined in Section 1.2.7, where HCl plays a crucial role in photo-oxidation of the matrix; a role that, until now, was not considered at all in commonly accepted theories of weathering.

Therefore, acid scavengers are beneficial to prevent weathering degradation, acting in several ways:

1. First, protecting the polymer during processing limits the generation of conjugated double bonds acting as chromophores that initiate photodegradation and photo-oxidation (Section 1.2.2 and Section 1.2.3).
2. Providing a reservoir of stabilizers limits the evolution of colored conjugated double bonds underskin in the absence of oxygen (Section 1.2.5)
3. Providing a reservoir of stabilizers limits the evolution of cracking, and eventually chalking, on the surface in the presence of oxygen (Section 1.2.7).

It is worth noting that not all acid scavengers have the same positive effect, but that some of them are neutral or even detrimental to surface discoloration, probably due to the water uptake stimulating the photo-oxidation of the matrix.

The design of the appropriate stabilizer formulation, thus, still requires a complex choice of proper ingredients and needs several laboratory and, eventually, real field tests.

## Figures and Tables

**Table 1:** Maxima of UV-vis absorption of polyene sequences (*n* = number of conjugated double bonds), ref. [19].

*n*	Isomer	Wavelength in nm									
3	Triene	240	268								
4	Tetraene		267	278	290	304					
5	Pentaene			279	290	304	317	334			
6	Hexaene					300	313	328	364		
7	Heptaene						316	332	368	390	
8	Octaene							332	367	386	410
	Average *	240	268	279	290	33	315	332	366	388	410

* Solar irradiation at Earth: 290–410 nm.

**Table 2:** Quantum yield of the primary process in the photodegradation of PVC (expressed as mmol/einstein^−1^), ref. [18].

Polymer Atmosphere	N_2_	O_2_
φ_HCl_ * _(= HCl evolution)_	11.0	15.0
φ_CS_ * _(= chain scission)_	3.1	2.6
φ_CL_ * _(= crosslinking)_	1.4	0.6
φ_>C=O_ * _(= carbonyls)_		5.0
φ_POOH_ * _(= hydroperoxides)_		3.0

* φ values expressed as mmol/einstein^−1^.

**Table 3:** Type and loading of tested acid scavengers vs. formulation numbers of Figure 26.

	1 Part *w*/*w*	4 Parts *w*/*w*	8 Parts *w*/*w*
Zeolite 4A ^1^	#1	#2	#3
Acid Scavenger One-Pack A ^2^	#4	#5	#6
Acid Scavenger One-Pack B ^3^	#9	#10	#11
Magnesium di-Hydroxide ^4^	#12	#13	#14
(Ca, Mg)(OH)_2_, SiO_2_ ^5^	#18	#19	#20

^1^ From PQ Silicas BV, Eijsden Netherlands. ^2^ Acid scavenger whose patent is pending. ^3^ Acid scavenger whose patent is pending. ^4^ Stearic acid coated ground milled brucite from Lehmann & Voss & Co., Hamburg, Germany. ^5^ Seastab 510 From Sea Water Chemical, Fukuoka, Japan comprises (Ca, Mg)(OH)_2_, SiO_2_ and may have, for example, about 67.2 weight percent CaO, 1.09 weight percent MgO, and 3.26 weight percent SiO_2_.

## Data Availability

The data presented in this study are available as Supplementary Materials.

## Supplementary Materials

The following supporting information can be downloaded at: https://www.mdpi.com/article/10.3390/polym16081047/s1, Table S1: Outdoor weathering data; Table S2: Xenon-arc tester data; Table S3: QUV Accelerated Weathering Tester. The data presented in this study are available as Supplementary material.
