# Supplementary material for: Surface Chalking upon Weathering of Dark-Colored PVC Articles and Relevant Stabilizers
Source: Polymers (Basel). 2024 Apr 10;16(8):1047. doi: 10.3390/polym16081047 (PMC11053820; doi:10.3390/polym16081047)
Supplement: Supplementary file 1 [file polymers-16-01047-s001.zip › polymers-2925520-supplementary.pdf]

TECHNICAL SERVICE LABORATORY

S. Giorgio di Piano, Bologna

Strictly confidential

Article

Surface Chalking upon Weathering of Dark-Colored PVC Articles and Relevant Stabilizers

Table S1. Outdoor weathering data

Sample	Parameter	0 months	4 months	6 months	12 months	18 months
1	L*	32.27	35.62	36.48	38.04	38.9
1	a*	-8.16	-9.32	-9.91	-11.59	-13.89
1	b*	0.81	-0.29	-0.43	0.6	0.58
1	ΔE	0	3.71	4.72	6.72	8.77
2	L*	32.31	35.67	36.3	37.33	37.1
2	a*	-8.3	-9.49	-10.21	-11.2	-13.42
2	b*	0.63	-0.37	-0.47	0.56	0.73
2	ΔE	0	3.70	4.56	5.80	7.01
3	L*	32.24	35.07	36.51	37.16	36.13
3	a*	-8.11	-9.39	-9.92	-10.54	-13.56
3	b*	0.63	-0.32	-0.39	0.46	0.8
3	ΔE	0	3.25	4.75	5.49	6.70
4	L*	32.23	34.75	35.72	36.81	38.97
4	a*	-8.24	-8.68	-9.07	-10.21	-12.83
4	b*	0.92	0.11	-0.04	0.58	0.42
4	ΔE	0	2.68	3.71	5.00	8.17
5	L*	33.05	33	34.42	35.02	36.4
5	a*	-8.1	-8.88	-8.91	-9.54	-10.39
5	b*	0.66	-0.03	-0.09	0.24	-0.33
5	ΔE	0	1.04	1.76	2.48	4.18
6	L*	32.62	33.37	33.91	34.87	35.79
6	a*	-8.12	-8.69	-8.92	-9.34	-11.6
6	b*	0.66	0.23	0.05	0.31	0.37
6	ΔE	0	1.04	1.64	2.58	4.72
9	L*	32.41	34.89	35.74	36.73	38.52
9	a*	-8.27	-8.78	-9.25	-10.41	-12.97
9	b*	0.78	0.02	-0.12	0.48	0.49
9	ΔE	0	2.64	3.59	4.83	7.71

strictly company confidential

10	L*	32.73	33.88	34.54	35.42	36.53
10	a*	-7.84	-8.63	-9.13	-9.87	-10.93
10	b*	0.89	0.13	0.01	0.5	0.18
10	ΔE	0	1.59	2.39	3.39	4.95
11	L*	32.29	32.45	33.08	34.3	34.54
11	a*	-7.84	-8.74	-8.89	-9.3	-9.47
11	b*	0.87	0.28	0.1	0.31	0.03
11	ΔE	0	1.09	1.52	2.55	2.90
12	L*	32.54	36.04	37.2	38.27	40.26
12	a*	-8.19	-9.38	-10.18	-11.92	-15.18
12	b*	0.85	-0.06	-0.15	0.9	0.63
12	ΔE	0	3.81	5.16	6.84	10.42
13	L*	32.29	35.6	36.59	37.88	40.68
13	a*	-8.14	-8.89	-9.68	-11.13	-15.13
13	b*	0.85	-0.06	-0.22	0.64	0.66
13	ΔE	0	3.51	4.69	6.34	10.92
14	L*	32.58	34.99	36.51	37.22	39.77
14	a*	-7.93	-8.82	-9.35	-10.72	-14.09
14	b*	0.78	-0.29	-0.44	0.52	0.41
14	ΔE	0	2.78	4.35	5.42	9.48
18	L*	32.88	36.2	37.25	38.44	37.23
18	a*	-8.12	-9.49	-10.43	-11.92	-12.43
18	b*	0.85	-0.06	-0.21	0.88	0.47
18	ΔE	0	3.71	5.06	6.73	6.14
19	L*	32.26	37.62	39.44	40.77	43.02
19	a*	-8.3	-9.86	-10.73	-12.82	-16.84
19	b*	0.97	-0.08	-0.17	1.07	1.53
19	ΔE	0	5.68	7.67	9.64	13.75
20	L*	32.47	37.88	39.5	41.81	40.89
20	a*	-8.38	-9.7	-10.57	-12.59	-14.75
20	b*	0.85	-0.14	-0.43	0.72	0.87
20	ΔE	0	5.66	7.47	10.25	10.56

strictly company confidential

Table S2. Xenon-arc tester data

Sample	Parameter	0 h	500 h	1000 h	1500 h	2000 h	2500 h	3000 h	3500 h	4000 h
1	L*	32.27	33.15	34.78	36.59	35.17	33.35	31.45	32.16	32.95
1	a*	-8.16	-8.75	-9.75	-11.13	-12.99	-17.42	-16.66	-19.43	-18.94
1	b*	0.81	0.03	0.42	0.77	1.49	1.97	2.06	2.88	2.94
1	ΔE	0	1.32	3.00	5.24	5.67	9.39	8.63	11.46	11.01
2	L*	32.31	33.63	36.38	34.73	34.03	33.65	32.87	32.57	33.15
2	a*	-8.3	-8.66	-10.15	-9.97	-13.44	-15.57	-17.66	-18.44	-18.85
2	b*	0.63	-0.07	-0.19	0.64	0.82	1.16	1.67	2.2	2.65
2	ΔE	0	1.54	4.55	2.94	5.42	7.41	9.43	10.26	10.77
3	L*	32.24	33.92	35.52	36.18	34.63	34.14	32.77	34.53	35.73
3	a*	-8.11	-8.5	-9.8	-11.16	-13.18	-17.2	-17.27	-19.27	-20.48
3	b*	0.63	0.05	0.17	0.43	0.95	1.08	1.23	1.53	2.09
3	ΔE	0	1.82	3.72	4.99	5.61	9.30	9.19	11.43	12.94
4	L*	32.23	33	34.81	36.27	36.71	34.31	31.26	32.84	32.77
4	a*	-8.24	-8.53	-9.37	-10.32	-11.44	-17.08	-17.13	-19.88	-18.89
4	b*	0.92	0.37	0.45	0.67	1.21	1.93	2.3	3.08	3.4
4	ΔE	0	0.99	2.86	4.55	5.51	9.14	9.05	11.85	10.95
5	L*	33.05	32.74	33.01	36.38	35.97	33.51	31.34	31.61	30.91
5	a*	-8.1	-8.64	-9.35	-9.87	-10.47	-14.09	-14.83	-17.46	-17.18
5	b*	0.66	0.33	0.06	0.03	0.6	1.25	1.56	2.31	2.85
5	ΔE	0	0.70	1.39	3.82	3.76	6.04	7.00	9.61	9.58
6	L*	32.62	33.17	33.79	35.66	35.35	33.3	30.79	31.25	30.12
6	a*	-8.12	-8.41	-9.02	-9.81	-10.84	-13.89	-15.2	-16.97	-16.85
6	b*	0.66	0.37	0.29	0.22	0.66	1.15	1.68	2.08	2.6
6	ΔE	0	0.69	1.52	3.51	3.85	5.83	7.38	9.07	9.29
9	L*	32.41	33.15	34.74	35.55	33.66	33.16	31.37	32.4	33.09
9	a*	-8.27	-8.55	-9.27	-10.36	-13.94	-18.58	-17.97	-19.26	-19.18
9	b*	0.78	0.34	0.51	0.77	1.64	2.2	2.57	3.15	3.61
9	ΔE	0	0.91	2.55	3.77	5.87	10.43	9.92	11.24	11.29

strictly company confidential

10	L*	32.73	32.46	34.59	35.1	33.51	31.93	31.07	31.21	30.75
10	a*	-7.84	-8.49	-9.14	-9.96	-11.89	-16.51	-15.97	-18.5	-18.36
10	b*	0.89	0.46	0.38	0.62	1.3	1.82	1.95	2.7	3.26
10	ΔE	0	0.82	2.33	3.19	4.14	8.76	8.37	10.92	10.96
11	L*	32.29	32.42	33.14	34.99	35.37	33.07	30.01	31.54	30.22
11	a*	-7.84	-8.37	-9.02	-9.79	-10.7	-13.98	-15.44	-16.73	-16.39
11	b*	0.87	0.5	0.44	0.36	0.74	1.25	1.8	2.14	2.74
11	ΔE	0	0.66	1.52	3.37	4.21	6.20	7.99	9.01	8.99
12	L*	32.54	33.51	35.78	35.64	34.3	34.84	33.48	33.71	34.39
12	a*	-8.19	-8.61	-9.82	-10.67	-15.01	-19.27	-18.61	-20.41	-20.83
12	b*	0.85	0.38	0.65	1.14	1.97	2.41	2.68	3.28	3.66
12	ΔE	0	1.16	3.63	3.98	7.13	11.42	10.62	12.51	13.08
13	L*	32.29	33.44	35.52	37.42	36.3	35.45	32.82	33.72	34.72
13	a*	-8.14	-8.53	-9.45	-11.06	-12.34	-17.58	-18.16	-20.25	-20.51
13	b*	0.85	0.38	0.5	0.69	1.68	1.9	2.59	3.2	3.39
13	ΔE	0	1.30	3.50	5.90	5.87	10.01	10.18	12.42	12.86
14	L*	32.58	33.11	35.29	36.42	36.19	34.38	34.18	34.89	33.99
14	a*	-7.93	-8.45	-9.53	-10.37	-13.2	-18.85	-20.09	-20.9	-20.05
14	b*	0.78	0.39	0.2	0.76	1.27	2.08	2.64	3.03	3.59
14	ΔE	0	0.84	3.20	4.55	6.41	11.14	12.41	13.36	12.52
18	L*	32.88	33.64	34.56	34.7	33.16	33.48	34.01	33.85	33.59
18	a*	-8.12	-8.77	-9.45	-10.55	-14.09	-18.47	-19.02	-19.8	-19.12
18	b*	0.85	0.37	0.7	1.06	1.94	2.35	2.58	3.19	3.26
18	ΔE	0	1.11	2.15	3.04	6.08	10.48	11.09	11.95	11.28
19	L*	32.26	34.86	37.35	38.44	37.31	36.03	35.13	35.68	35.83
19	a*	-8.3	-8.9	-10.5	-11.97	-15.59	-19.06	-18.6	-20.76	-20.22
19	b*	0.97	0.39	0.58	0.97	2.05	2.56	2.64	3.57	3.86
19	ΔE	0	2.73	5.56	7.19	8.93	11.51	10.82	13.18	12.77
20	L*	32.47	33.52	37.77	38.43	38.39	37.37	33.83	34.13	36.37
20	a*	-8.38	-9.33	-10.78	-12.3	-14.84	-20.05	-18.2	-20.98	-20.5
20	b*	0.85	0.75	0.4	1.02	1.93	2.24	2.62	3.8	4.46
20	ΔE	0	1.42	5.84	7.14	8.83	12.73	10.07	13.05	13.23

tly company confidential

Table S3. QUV Accelerated Weathering Tester

Sample	Parameter	0 h	300 h	600 h	900 h	1200 h	1500 h	1800 h	2100 h	2400 h	2700 h	3000 h	3300 h	3600 h	3900 h	4200 h
1	L*	32.27	31.53	37.75	35.50	38.77	37.99	38.00	37.99	37.49	38.44	38.46	38.73	37.9	38.61	38.62
1	a*	-8.16	-8.58	-8.66	-9.58	-10.42	-10.90	-11.73	-12.96	-12.78	-12.73	-12.42	-13.36	-14	-13.92	-13.88
1	b*	0.81	0.18	-1.10	-0.29	-0.50	0.60	0.63	0.78	1.44	1.03	1.43	1.12	1.33	1.65	1.72
1	ΔE	0.00	1.06	5.82	3.70	7.01	6.35	6.75	7.47	7.00	7.68	7.54	8.30	8.13	8.61	8.59
2	L*	32.99	31.74	35.35	39.8	39.15	39.11	38.29	37.1	37.21	37.95	37.96	37.98	39.94	39.5	38.03
2	a*	-8.03	-8.82	-8.96	-10.87	-10.76	-11.4	-12.36	-12.48	-12.88	-13.41	-13.33	-12.3	-13.08	-12.09	-13.23
2	b*	0.61	0.02	-1.33	-1.54	-0.19	0.23	0.7	0.16	0.93	0.93	1.11	1.16	1.39	1.53	1.84
2	ΔE	0	1.59	3.19	7.69	6.79	7.00	6.84	6.07	6.44	7.32	7.28	6.59	8.63	7.73	7.35
3	L*	32.24	32.11	33.32	34.75	37.27	37.74	38.65	38.87	38.07	38.47	38.83	39.1	39.31	39.43	39.72
3	a*	-8.11	-8.40	-8.86	-9.24	-10.00	-10.59	-10.91	-11.80	-12.52	-13.6	-12.85	-13.3	-13.63	-14.11	-14.18
3	b*	0.63	0.17	-0.35	-0.38	-0.26	0.54	0.32	0.39	0.86	0.58	0.27	0.57	0.91	1.27	1.58
3	ΔE	0.00	0.56	1.64	2.93	5.45	6.03	7.00	7.59	7.31	8.30	8.13	8.60	8.97	9.39	9.68
4	L*	32.23	32.24	33.52	34.53	38.20	37.07	37.70	38.97	37.35	38.31	38.75	38.52	39.09	38.84	38.77
4	a*	-8.24	-8.26	-8.35	-8.88	-9.33	-9.77	-10.05	-11.92	-12.45	-13.06	-13.21	-13.39	-13.37	-13.69	-14.05
4	b*	0.92	0.52	-0.18	-0.40	-0.47	0.23	0.49	0.50	1.25	1.09	1.05	1.2	1.44	1.68	2.02
4	ΔE	0.00	0.40	1.70	2.73	6.23	5.12	5.78	7.69	6.64	7.76	8.20	8.13	8.58	8.60	8.82
5	L*	33.05	31.66	32.14	33.78	38.71	37.42	39.21	39.60	37.59	37.96	37.7	38.17	36.74	39.29	39.21
5	a*	-8.10	-8.67	-8.58	-8.51	-8.67	-9.14	-9.45	-10.28	-10.34	-12.05	-12.16	-13.45	-12.61	-12.36	-11.83
5	b*	0.66	0.37	0.15	-0.85	-1.56	-0.67	-0.50	-0.44	0.05	0.4	0.27	0.84	1.48	1.03	1.23
5	ΔE	0.00	1.53	1.15	1.73	6.11	4.68	6.41	6.99	5.10	6.31	6.19	7.41	5.88	7.56	7.22
6	L*	32.62	32.55	32.73	33.73	39.87	36.53	38.91	41.42	38.81	39.02	39.1	38.62	39.16	39.62	39.42
6	a*	-8.12	-8.15	-8.30	-8.26	-8.23	-8.78	-9.57	-10.42	-9.98	-11.74	-12.17	-12.7	-12.37	-11.57	-11.3
6	b*	0.66	0.45	0.36	0.01	-1.63	-0.35	-0.75	-0.88	-0.1	-0.02	0.15	0.37	0.12	0.18	0.3
6	ΔE	0.00	0.22	0.37	1.29	7.60	4.09	6.61	9.23	6.51	7.38	7.66	7.55	7.82	7.82	7.52
9	L*	32.41	32.47	33.61	40.48	43.82	38.78	39.88	40.80	40.22	40.73	40.51	39.46	39.56	39.58	39.71
9	a*	-8.27	-8.32	-8.40	-8.50	-10.31	-9.97	-11.29	-12.49	-12.53	-14.6	-14.23	-15.12	-14.36	-14.27	-13.88
9	b*	0.78	0.48	-0.16	-1.03	-1.45	-0.13	-0.12	-0.12	0.51	0.63	0.52	1.05	0.84	1.05	1.06

tly company confidential

9	ΔE	0.00	0.31	1.53	8.27	11.80	6.66	8.11	9.43	8.90	10.46	10.06	9.83	9.39	9.35	9.21
10	L*	33.01	32.02	32.97	33.75	36.98	39.94	39.71	35.42	38.68	38.99	38.22	37.53	38.68	34.58	35.9
10	a*	-7.66	-8.42	-8.4	-8.78	-9.19	-10.27	-11.1	-10.45	-13.5	-13.6	-13.02	-13.09	-12.48	-11.42	-11.12
10	b*	0.85	0.46	-0.07	-0.16	-0.34	-0.5	-0.06	0.39	0.83	0.74	1.08	1.11	0.99	2.62	2.19
10	ΔE	0	1.31	1.18	1.68	4.42	7.53	7.59	3.72	8.14	8.43	7.48	7.07	7.44	4.44	4.70
11	L*	32.29	31.96	32.03	32.79	38.91	34.92	37.19	38.13	39.62	39.75	39.44	39.15	39.62	39.86	37.19
11	a*	-7.84	-8.13	-8.25	-8.23	-8.25	-9.09	-9.51	-9.92	-10.27	-11.77	-12.35	-12.65	-12.22	-11.97	-12.5
11	b*	0.87	0.55	0.41	0.02	-1.55	-0.19	-0.42	-0.04	-0.16	0.13	0.35	0.44	0.51	0.72	0.82
11	ΔE	0.00	0.54	0.67	1.06	7.06	3.10	5.34	6.27	7.79	8.46	8.47	8.39	8.55	8.62	6.76
12	L*	32.54	32.57	36.23	38.66	40.66	40.35	40.42	39.72	39.58	40.49	40.1	39.25	39.51	39.22	39.14
12	a*	-8.19	-8.42	-8.61	-9.84	-10.92	-12.01	-12.44	-14.12	-14.09	-14.85	-14.11	-15.12	-15.17	-14.96	-15.4
12	b*	0.85	0.57	-0.78	-0.86	-0.50	0.25	0.61	1.03	1.11	0.86	0.9	2.19	2.46	2.09	2.32
12	ΔE	0.00	0.36	4.06	6.57	8.67	8.71	8.96	9.31	9.19	10.37	9.60	9.74	9.99	9.59	9.88
13	L*	32.29	32.57	34.30	40.04	40.15	38.38	37.93	39.46	39.97	41.2	41.02	40.76	41.2	40.91	40.7
13	a*	-8.14	-8.28	-8.38	-9.01	-9.93	-10.51	-10.98	-13.02	-12.88	-12.99	-12.31	-12.58	-13.53	-13.38	-13.71
13	b*	0.85	0.51	-0.36	-1.47	-0.98	0.35	0.69	0.73	0.51	0.74	0.97	1.8	1.99	2.41	2.54
13	ΔE	0.00	0.46	2.36	8.14	8.27	6.55	6.32	8.67	9.03	10.15	9.68	9.61	10.48	10.21	10.23
14	L*	32.58	32.90	34.31	36.97	38.33	37.58	37.63	38.44	38.95	39.42	39.35	41.02	42	40.95	41.2
14	a*	-7.93	-8.17	-8.43	-8.98	-9.35	-10.50	-11.58	-12.44	-13.34	-14.83	-15.22	-13.13	-13.33	-14.25	-14.16
14	b*	0.78	0.35	-0.48	-1.38	-1.08	0.24	0.78	0.36	0.69	1.19	1.35	2.25	2.62	2.21	2.55
14	ΔE	0.00	0.59	2.20	5.00	6.21	5.65	6.23	7.41	8.36	9.72	9.97	10.02	11.01	10.59	10.78
18	L*	32.88	32.18	34.74	32.90	36.06	37.47	36.99	38.17	37.83	38.46	38.74	39.07	39.3	38.33	38.5
18	a*	-8.12	-8.53	-8.66	-9.76	-9.63	-10.85	-11.69	-12.83	-14.11	-14.92	-13.8	-13.35	-13.48	-14.55	-14.74
18	b*	0.85	0.45	-0.18	0.12	0.14	0.91	1.05	0.86	1.06	1.33	0.83	1.61	1.93	2.3	2.5
18	ΔE	0.00	0.90	2.19	1.80	3.59	5.34	5.45	7.08	7.77	8.81	8.16	8.14	8.43	8.55	8.84
19	L*	32.71	33.81	38.02	39.89	40.56										
19	a*	-8.1	-8.58	-9.21	-10.25	-11.22										
19	b*	0.93	0.27	-1	-0.32	0.31										
19	ΔE	0	1.37	5.76	7.60	8.47										
20	L*	32.47	33.22	41.46	42.03	41.59	43.72	40.81	42.18	41.35	42.29	42.05	42.8	43.25	42.88	43.41
20	a*	-8.38	-8.63	-8.79	-10.16	-10.90	-13.13	-13.46	-15.26	-16.72	-17.67	-17.32	-16.72	-15.97	-15.98	-15.45

tly company confidential

20	b*	0.85	0.54	-1.64	-1.29	-0.33	0.52	1.15	0.83	1.16	1.5	1.79	2.9	3.29	3.21	3.62
20	ΔE	0.00	0.85	9.34	9.96	9.54	12.22	9.77	11.90	12.19	13.53	13.14	13.43	13.41	13.10	13.32

Strictly company confidential
